# Effects of a simple cardiac rehabilitation program on improvement of self-reported physical activity in atrial fibrillation – Data from the RACE 3 study

**DOI:** 10.1016/j.ijcha.2020.100673

**Published:** 2020-11-16

**Authors:** Bao Oanh Nguyen, E.P.J. Petra Wijtvliet, Anne H. Hobbelt, Simone I.M. De Vries, Marcelle D. Smit, Robert G. Tieleman, Dirk Jan Van Veldhuisen, Harry J.G.M. Crijns, Isabelle C. Van Gelder, Michiel Rienstra, for the RACE 3 Investigators

**Affiliations:** a Department of Cardiology, University Medical Center Groningen, University of Groningen, Groningen, the Netherlands; b Martini Hospital, Groningen, the Netherlands; c Maastricht University Medical Center+ and Cardiovascular Research Institute Maastricht, Maastricht, the Netherlands

**Keywords:** Atrial Fibrillation, Physical activity, Cardiac rehabilitation

## Abstract

**Background and aim:**

Physical inactivity is associated with an increased prevalence of atrial fibrillation (AF). We aim to evaluate whether cardiac rehabilitation (CR) motivates patients to become and stay physical active, and whether CR affects sinus rhythm maintenance and quality of life (QoL) in patients with persistent AF and moderate heart failure.

**Methods:**

In the Routine versus Aggressive risk factor driven upstream rhythm Control for prevention of Early atrial fibrillation in heart failure study patients were randomized to conventional or targeted therapy. Targeted therapy contained next to optimal risk factor management a 3-month CR program, including self-reported physical activity and counseling. Successful physical activity was assessed in the targeted group, defined as activity of moderate intensity ≥ 150 min/week, or ≥ 75 min/week of vigorous intensity. AF was assessed at 1 year on 7-days Holter monitoring, QoL using general health, fatigue and AF symptom questionnaires.

**Results:**

All 119 patients within the targeted group participated in the CR program, 106 (89%) completed it. At baseline 80 (67%) patients were successfully physical active, 39 (33%) were not. NTproBNP was lower in active patients. During 1-year follow-up physical active patients stayed active: 72 (90%) at 12 weeks, 72 (90%) at 1 year. Inactive patients became active: at 12 weeks 25 (64%) patients and 30 (77%) at 1 year. No benefits were seen on sinus rhythm maintenance and QoL for successful physical active patients.

**Conclusion:**

In patients with persistent AF and moderate heart failure participation in CR contributes to improve and to maintain physical activity.

## 1 Introduction

Atrial fibrillation (AF) is associated with an increased risk of cardiovascular morbidity and mortality [1]. Underlying conditions such as diabetes, hypertension, coronary artery disease and obesity cause atrial remodeling, resulting in AF progression. Physical activity has been shown to prevent underlying comorbidities and lower the risk of AF [2]. Low exercise capacity is associated with an increased risk of mortality and cardiovascular hospitalization and improvement of exercise capacity lowers these risks [3], [4]. Therefore the guidelines recommend moderate regular physical activity to prevent AF [[1], [2], [5]]. It has been shown that health benefit is gained with > 150 min moderate intensity activity per week, or 75 min vigorous intensity, or a combination, and that this strategy may reduce the risk of incident AF by 10% [6]. In addition, exercise training was associated with an improvement quality of life (QoL) [7]. In the CARDIOrespiratory FITness on Arrhythmia Recurrence in Obese Individuals With Atrial Fibrillation (CARDIO-FIT) trial the combination of risk factor management and an exercise program reduced the recurrence of AF [8].

In the Routine versus Aggressive risk factor driven upstream rhythm Control for prevention of Early AF in heart failure (RACE 3) trial persistent AF patients were randomized to targeted or routine therapy. Patients in the targeted group received statins, mineralocorticoid receptor antagonists (MRA), angiotensin-converting enzyme inhibitors (ACE-inhibitors) and/or angiotensin receptor blockers (ARB) and a 3-month cardiac rehabilitation (CR) program including physical therapy and counseling [9].

We hypothesize that CR motivates patients to become more physical active, and consequently improves underlying conditions of AF, maintenance of sinus rhythm and QoL. Therefore, our aim is to evaluate whether CR motivates patients to become and stay physically active, and whether CR affects sinus rhythm maintenance and QoL in patients with persistent AF and moderate heart failure (HF) included in the RACE 3 trial.

## 2 Methods

### 2.1 Study design

The study design has been published previously [9], [10]. Briefly, the RACE 3 (Clinicaltrials.gov identifier NCT00877643) was a prospective, randomized, open-label, multicenter trial in patients with early persistent AF and mild to moderate HF. The study was performed in compliance of the Declaration of Helsinki. The Institutional Review Board of all participating hospitals approved the study, and all patients gave written informed consent. Patients were randomized to targeted therapy of underlying conditions or conventional therapy. The targeted therapy group patients received four therapies on top of routine therapy: (1) MRAs, (2) statins, (3) ACE-inhibitors and/or ARBs, and (4) CR.

The CR program started immediately after inclusion. During CR supervised physical activity took place 2 to 3 times per week and lasted 9 to 11 weeks. Completion of the CR program was defined as following the program for a minimum of 8 weeks. Counseling by a nurse took place once every 6 weeks, starting 1 week after inclusion, continuing to end of study at 1 year. During counseling patients were encouraged to improve lifestyle and stimulated to exercise on a regular basis, on a moderate level. Physical activity was evaluated in the targeted therapy group according to physical achievement at every counseling visit and documented in the case record form. Physical activity was patient tailored and included sports, walking, biking and lower intensity exercise such as vacuuming and gardening. The endpoint was achievement of successful physical activity at 1 year, defined as performing physical activity a minimum of 150 min per week on a moderate intensity (3–6 METs), or a minimum of 75 min of vigorous intensity (> 6 METs).

Sinus rhythm maintenance was assessed on a 7-day Holter. Quality of life was assessed by Medical Outcomes Study Short-Form Health Survey (SF-36) questionnaire, the University of Toronto AF severity scale part C and the Multidimensional Fatigue Index. The SF-36 questionnaire consist of 36 questions to calculate eight scales. Scores from each scale were translated to a score from 0 to 100, with a score of 100 indicating the best QoL [11]. In the AFSS part C questionnaire questions are scored 0 to 5, with a possible total score of 0 to 35. High scores indicates more AF-related symptoms [12]. The MFI-20 consists of 20 questions. In the current study, questions were scored from 1 to 6, making scale scores from 4 to 24, with higher scores indicating more fatigue [13].

### 2.2 Statistical analysis

Baseline characteristics are presented as mean ± standard deviation (SD) for normally distributed data, as median and interquartile range for non-normally distributed continuous data, and as number of patients and percentage for categorical data. Analyses were conducted with IBM SPSS statics version 23 or higher. The Chi-square, Fisher’s exact or Mann-Whitney *U* test were used for between group differences. The McNemar and Wilcoxon signed-rank test were used for within group analysis. A P-value of <0.05 was considered statistically significant.

## 3 Results

**Fig. 1:**
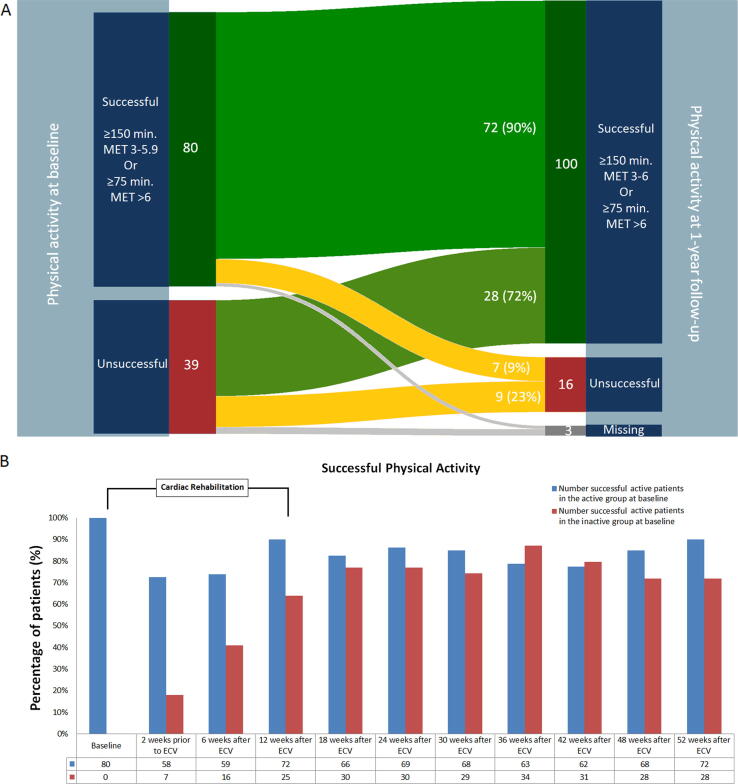
Successful Physical Activity. A. Patients categorization based on successful and unsuccessful physical activity at baseline (left) and 1-year follow-up (right). Min., minutes; MET, metabolic equivalent. B. Patients who were successful physically active during follow-up in those who were successfully active (active group) and those who were not active at baseline (inactive group). Blue bars: % patients who were successful active at baseline and stayed successful active. Red bars: % patients who were unsuccessful active who became physically active. (For interpretation of the references to colour in this figure legend, the reader is referred to the web version of this article.)

All 119 patients with AF and moderate HF randomized to targeted therapy participated in the CR program. Baseline characteristics between physically active and inactive patients were comparable, except for NTproBNP (946 (597–1403) versus 1305 (820–216) pg/mL, *p* = 0.005, respectively). Mean age was 64 ± 9, 94 (79%) were men, hypertension was present in 66 (55%), HF with preserved ejection fraction in 84 (71%) patients (Table 1). At baseline 80 (67%) were successful physically active. The total number of successful physically active patients increased during CR to 95 (82%) at 12 weeks (*p* = 0.005), and 100 (86%) patients at 1 year (*p* = 0.001) (Fig. 1A).

**Table 1:** Baseline characteristics.

Characteristic	Total (n = 119)	Successful physical active patients (n = 80)	Unsuccessful physical active patients (n = 39)	P-value
Age (years)	64 ± 9	65 ± 8	64 ± 9	0.713
Male sex	94 (79%)	66 (83%)	28 (72%)	0.231
Total duration AF (months)	3 (2–7)	4 (2–7)	3 (2–6)	0.535
Total persistent AF (months)	2 (1–4)	2 (1–4)	2 (2–3)	0.279
Duration heart failure (months)	2 (1–4)	2 (1–4)	2 (2–4)	0.179
Hospital admission for HF	14 (12%)	8 (10%)	6 (15%)	0.383
Hypertension	66 (55%)	45 (56%)	21 (54%)	0.846
Diabetes	10 (8%)	8 (10%)	2 (5%)	0.495
Coronary artery disease	19 (16%)	13 (16%)	6 (15%)	1.000
Ischemic thromboembolic complications	6 (5%)	4 (5%)	2 (5%)	1.000
Chronic obstructive pulmonary disease	9 (8%)	7 (9%)	2 (5%)	0.716
CHA_2_DS_2_-VASc score*	2 (1–3)	2 (1–3)	2 (1–3)	0.767
Body mass index (kg/m^2^)	29 (26–31)	28 (26–27)	30 (27–32)	0.136
Blood pressure (mmHg)				
Systolic	130 ± 15	129 ± 16	134 ± 15	0.146
Diastolic	83 ± 10	83 ± 10	83 ± 12	0.984
EHRA class	2 (2–2)	2 (2–2)	2 (2–2)	0.074
NYHA classification				0.876
I	28 (24%)	18 (23%)	10 (26%)	
II	80 (67%)	54 (68%)	26 (67%)	
III	11 (9%)	8 (10%)	3 (8%)	
NTproBNP (pg/mL)	1052 (698–1694)	945.5 (597–1403)	1305 (820–2160)	0.005
Urine sodium (mmol/24 h)	1160 (119–206)	151 (108–199)	180 (94–197)	0.283
Medication				
Beta-blocker	102 (86%)	67 (84%)	35 (90%)	0.578
Verapamil/diltiazem	3 (3%)	2 (3%)	1 (3%)	1.000
Digoxin	32 (27%)	18 (23%)	14 (36%)	0.130
ACE-inhibitor	38 (32%)	26 (33%)	12 (31%)	1.000
Angiotensin receptor blocker	24 (20%)	16 (20%)	8 (21%)	1.000
Mineralocorticoid receptor antagonist	1 (1%)	1 (1%)	0 (0%)	1.000
Statin	40 (34%)	27 (34%)	13 (33%)	1.000
Diuretic	51 (43%)	31 (39%)	20 (51%)	0.238
Anticoagulant	116 (97%)	78 (98%)	38 (97%)	1.000
Echocardiographic variables				
Left atrial size, long axis (mm)	43 (40–48)	43 (40–47)	45 (40–48)	0.496
Left atrial volume indexed (mL/m^2^)	38 (31–48)	37 (31–46)	41 (28–53)	0.607
LV ejection fraction (%)	50 (43–58)	51 (43–56)	50 (43–60)	0.827
Exercise Test				
Maximum load (W)	134 (105–163)	140 (110–175)	126 (100–151)	0.080

Data are mean ± SD, number of patients (%) or median (IQR). ACE, angiotensin-converting enzyme; AF, atrial fibrillation; EHRA, European Heart Rhythm Association class for symptoms; HF, heart failure; LV, left ventricular; NT-proBNP, N-terminal pro-brain natriuretic peptide, NYHA, New York Heart Association.

* The CHA2DS2-VASc score assesses thromboembolic risk. C = congestive heart failure/LV dysfunction, H = hypertension; A2 = age ≥ 75 years; D = diabetes mellitus; S2 = stroke/transient ischemic attack/systemic embolism; V = vascular disease; A = age 65–74 years; Sc = sex category (female sex).

One-hundred-six (89%) patients completed the CR program. In those who completed the program there was a significant increase in successful physically active patients (73 [69%] to 93 patients [88%], *p* < 0.001) between baseline and 1 year. No difference was seen in those who did not complete CR. Physically active patients stayed active: 72 (90%) at 12 weeks and 72 (90%) at 1 year (Fig. 1B). Inactive patients became active: at 12 weeks 25 (64%) patients and 28 (72%) at 1 year (Fig. 1B). At 1 year sinus rhythm was maintained in 77 of 100 (77%) successful physically active versus 9 of 16 (56%) inactive patients (*p* = 0.120).

Between baseline and 1-year follow-up, both groups improved in the majority of the SF-36, AFSS and MFI subscales. No differences were seen between the successful physically active and inactive patients in the SF-36 subscales at 1-year follow-up. The AFSS subscale fatigue at rest changes was significantly more in the inactive patients (Δ-0.5 ± 1.26 versus Δ-1.17 ± 1.65, *p* = 0.027) compared to the successful active patients. The inactive patients at baseline improved more in general physical fatigue (Δ-2.47 ± 6.24 versus Δ-4.72 ± 4.80, *p* = 0.031) and mental fatigue (Δ-0.34 ± 4.41 versus Δ-1.97 ± 2.92, *p* = 0.047) between baseline and 1-year follow-up (Table 2).

**Table 2:** Changes in QoL.

	Physical activity	Baseline	1-year follow-up	P-value Within- group change	P-value Between- group change
SF-36 scores					
Physical functioning	Successful	70 ± 21	80 ± 22	<0.001	
	Unsuccessful	62 ± 25	78 ± 18	<0.001	0.162
Physical role limitations	Successful	45 ± 45	71 ± 39	<0.001	
	Unsuccessful	40 ± 41	80 ± 35	<0.001	0.216
Bodily pain	Successful	81 ± 23	85 ± 22	0.213	
	Unsuccessful	79 ± 20	87 ± 18	0.062	0.064
General health	Successful	61 ± 19	66 ± 22	<0.001	
	Unsuccessful	54 ± 17	65 ± 16	0.002	0.428
Vitality	Successful	61 ± 24	67 ± 21	0.001	
	Unsuccessful	53 ± 20	63 ± 18	0.011	0.305
Social functioning	Successful	77 ± 24	84 ± 20	0.015	
	Unsuccessful	79 ± 24	90 ± 17	0.024	0.356
Emotional role limitations	Successful	75 ± 40	82 ± 34	0.077	
	Unsuccessful	74 ± 41	88 ± 25	0.027	0.587
Mental health	Successful	79 ± 18	83 ± 15	0.031	
	Unsuccessful	77 ± 14	83 ± 14	0.013	0.238
AFSS scores					
Palpitations	Successful	1.4 ± 1.5	0.5 ± 0.8	<0.001	
	Unsuccessful	1.6 ± 1.5	0.5 ± 0.8	0.003	0.400
Dyspnoea at rest	Successful	1.2 ± 1.2	0.6 ± 0.9	<0.001	
	Unsuccessful	1.6 ± 1.5	0.5 ± 0.8	0.001	0.294
Dyspnoea during exercise	Successful	2.4 ± 1.4	1.3 ± 1.3	<0.001	
	Unsuccessful	2.7 ± 1.4	1.6 ± 1.5	0.002	0.960
Reduced exercise capacity	Successful	1.9 ± 1.4	1.0 ± 1.1	<0.001	
	Unsuccessful	2.7 ± 1.5	1.4 ± 1.4	<0.001	0.126
Fatigue at rest	Successful	1.3 ± 1.4	0.8 ± 1.1	0.002	
	Unsuccessful	1.9 ± 1.5	0.7 ± 0.9	0.001	0.027
Dizziness	Successful	0.8 ± 1.1	0.8 ± 1.1	0.664	
	Unsuccessful	1.2 ± 1.4	0.8 ± 1.0	0.098	0.193
Chest pain	Successful	0.7 ± 1.1	0.3 ± 0.6	0.002	
	Unsuccessful	0.4 ± 0.9	0.2 ± 0.5	0.301	0.242
MFI-20 scores					
General fatigue	Successful	14 ± 7	12 ± 6	0.001	
	Unsuccessful	17 ± 5	12 ± 5	<0.001	0.031
Physical fatigue	Successful	14 ± 6	11 ± 5	<0.001	
	Unsuccessful	17 ± 4	12 ± 5	<0.001	0.051
Reduced activity	Successful	14 ± 6	11 ± 5	<0.001	
	Unsuccessful	16 ± 4	12 ± 5	<0.001	0.179
Reduced motivation	Successful	11 ± 6	10 ± 5	0.031	
	Unsuccessful	13 ± 5	11 ± 4	0.006	0.069
Mental fatigue	Successful	10 ± 6	9 ± 5	0.517	
	Unsuccessful	10 ± 5	8 ± 5	0.002	0.047

Data are mean ± SD; SF-36, Medical Outcomes Study Short-Form Health Survey; AFSS, the University of Toronto AF severity scale; MFI-20, Multidimensional Fatigue Index.

## 4 Discussion

We studied if a relatively simple CR program can improve physical activity and consequently maintenance of sinus rhythm and QoL in patients with persistent AF and moderate HF. We show that CR increases the total number of patients performing successful physical activity, included in the targeted therapy group. This was especially due to more inactive patients who became and stayed successful physically active. We did not observe any benefit in maintenance of sinus rhythm nor in QoL in the successful physically active patients compared to inactive patients at 1 year follow-up.

In contrast to a more aggressive CR program, [8] we now show that an easy to implement, short term CR program followed by counseling thereafter is effective in motivating patients to become more physically active, and to sustain this change in lifestyle.

At 1-year follow-up no difference were observed in maintenance of sinus rhythm between the successful physically active patients and the inactive ones. This might predominantly be due to the small number of patients who were inactive at 1 year. Further, in contrast to previous studies our patients did not show significant weight reduction eliminating its additional beneficial effects on sinus rhythm maintenance [8], [14].

QoL improved in both groups at 1-year follow-up. The inactive patients improved slightly more in subscales fatigue at rest, general physical fatigue and mental fatigue, than the successful physically active patients. This most likely resulted from poorer scores at baseline. On top of that, the majority of the inactive patients became physically active during the CR program which also may explain the larger improvement in general fatigue and mental fatigue. Previous studies involving an exercise programme in highly motivated obese AF patients showed promising results in reduction of AF burden and improvement of QoL [8], [15], [16]. In contrast to our study, these were comprehensive CR programs. Furthermore, it may also be related to the small group of inactive patients at 1-year follow-up.

Limitations include the small number of patients, due to the lack of data on physical activity in the conventional group, the observational comparison, the self-reported physical activity, the absence of objective assessment of daily exercise and short follow-up.

## 5 Conclusion

Patients with persistent AF and mild to moderate HF stay physically active with an easy to implement CR program. There were no beneficial effects in maintenance of sinus rhythm or QoL for physically active patients.

Funding

The study is supported by the Netherlands Heart Foundation (Grant 2008B035).

Unrestricted grants from AstraZeneca, Bayer, Biotronik, Boehringer-Ingelheim, Boston Scientific, Medtronic, Sanofi-Aventis, St Jude Medical paid to the Netherlands Heart Institute. Dr. Tieleman reports grants and personal fees from Bayer, Bristol-Myers-Squibb, Pfizer, and Daiichi-Sankyo. All other authors have no competing interests.

## Declaration of Competing Interest

Dr. Tieleman reports grants and personal fees from Bayer, Bristol-Myers-Squibb, Pfizer, and Daiichi-Sankyo. All other authors report no relationships that could be construed as a conflict of interest.
